# Case Report: Therapeutic Strategy With Delayed Debridement for Culture-Negative Invasive Group A Streptococcal Infections Diagnosed by Metagenomic Next-Generation Sequencing

**DOI:** 10.3389/fpubh.2022.899077

**Published:** 2022-05-11

**Authors:** Wenfang He, Chenfang Wu, Yanjun Zhong, Jinxiu Li, Guyi Wang, Bo Yu, Ping Xu, Yiwen Xiao, Tiantian Tang

**Affiliations:** ^1^Department of Critical Care Medicine, The Second Xiangya Hospital, Central South University, Changsha, China; ^2^Department of Pharmacy, The Second Xiangya Hospital, Central South University, Changsha, China; ^3^Institute of Clinical Pharmacy, Central South University, Changsha, China; ^4^Hunan Provincial Engineering Research Centre of Translational Medicine and Innovative Drug, Changsha, China

**Keywords:** invasive group A streptococcal infections, streptococcal toxic shock syndrome, clindamycin, intravenous immunoglobulin, next-generation sequencing

## Abstract

Streptococcal toxic shock syndrome (STSS) caused by group A streptococcus is a rare condition that rapidly developed to multiple organ failure even death. Therefore, prompt diagnosis, initiate appropriate antibiotics and other supportive treatments are critical. Here we reported a case of STSS caused by group A streptococcus infection. A healthy 39-year-old man presented a sudden pain in the left lower extremity, followed by a high fever (40.0 °C) with dizziness, nausea, and shortness of breath. Twenty-four hours before the visit, the patient showed anuria. The patient was then admitted to the intensive care unit. Blood examination revealed elevated levels of inflammatory markers and creatinine. He suffered from septic shock, dysfunction of coagulation, acute kidney dysfunction, acute respiratory distress syndrome, and acute liver function injury. The diagnosis was obtained through clinical manifestation and metagenomic next-generation sequencing (mNGS) drawn from the pustule and deep soft tissue (lower limb) samples while all bacterial cultures came back negative. The pustule mNGS report detected a total of 132 unique group A streptococcus sequence reads, representing 96.3% of microbial reads while the soft tissue mNGS report identified a total of 142474 unique group A streptococcus sequence reads, representing 100% of microbial reads. The patient was treated with aggressive fluid resuscitation, antibiotics comprising piperacillin/tazobactam and clindamycin, respiratory support, following the delayed surgical debridement. Intravenous immunoglobulin was also used for 5 days. On the 14th day after admission, he was transferred to the general ward for follow-up treatment. Our case highlighted, for the first time, the key role of mNGS in the early diagnosis of culture-negative invasive group A streptococcal infection. The case also suggested that clindamycin combined with beta-lactam antibiotics and adjunction of intravenous immunoglobulin therapy with delayed debridement performed well in the management of unstable STSS patients.

## Introduction

Streptococcal toxic shock syndrome (STSS) is a rare but life-threatening disease following invasive group A streptococcal infections (iGAS). It is important to know the diagnostic criteria for this syndrome and its various spectra of presentation. STSS may occur in any site of infection but most commonly be seen in cutaneous lesions. Characteristics of iGAS include signs of toxicity and a rapidly progressing clinical course. Although group A streptococcus (GAS) is always sensitive to penicillin, mortality of iGAS with STSS is up to 38% (1). Treatment of STSS requires fluid resuscitation, appropriate antibiotics, and supportive care in the intensive care unit (ICU), usually involving invasive/non-invasive mechanical ventilation. Clindamycin combined with beta-lactam therapy may perform better than single antibiotics. Intravenous immunoglobulin is also recommended for the treatment of patients with STSS. Here we reported the metagenomic next-generation sequencing used for the first time in the diagnosis of iGAS with STSS. We aimed to summarize how to achieve the definite diagnosis of iGAS and manage it effectively.

## Case Description

**Figure 1:**
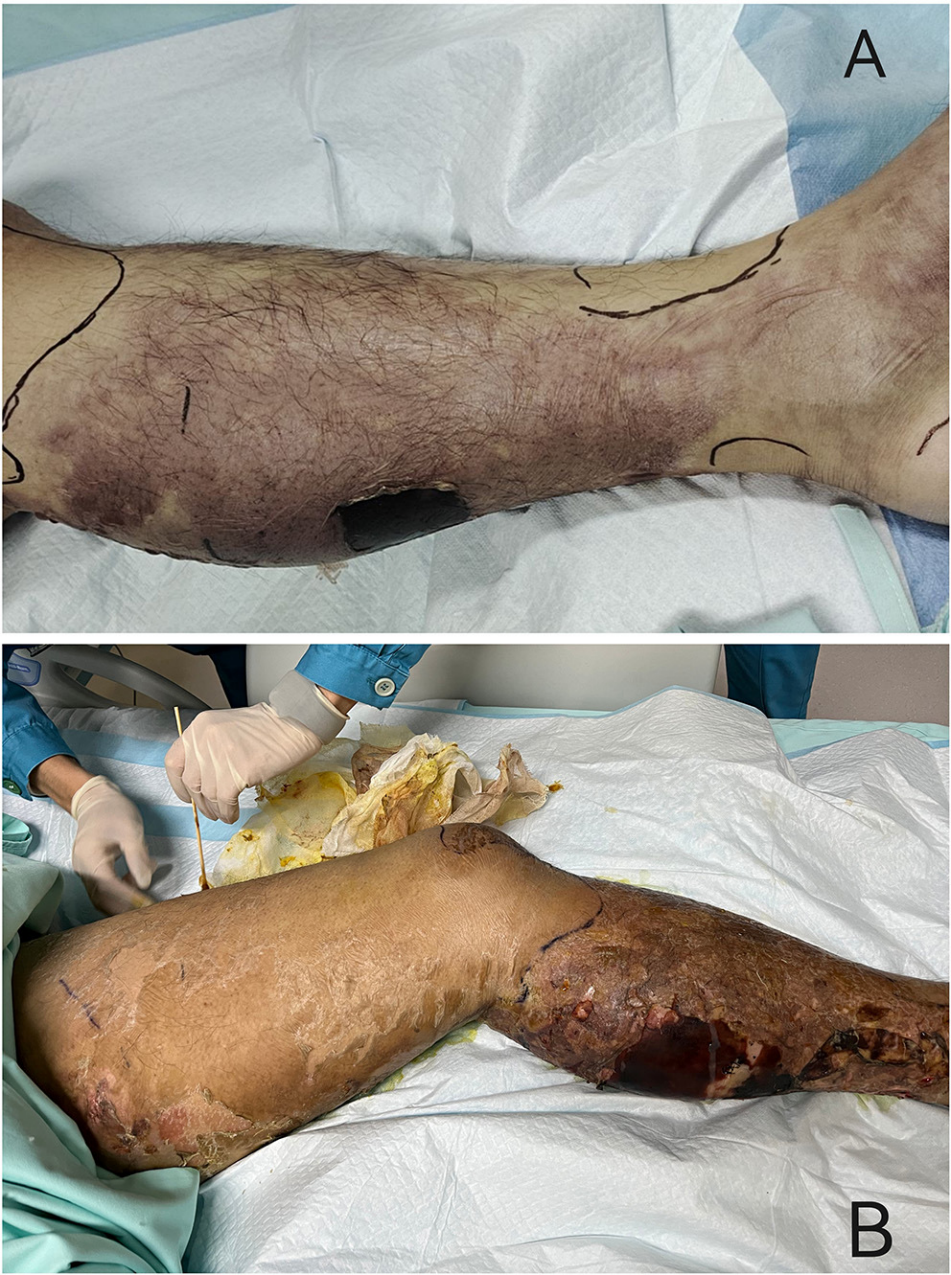
Clinical presentation of the patient. **(A)** At the admission to ICU, it could be seen the moderate swelling, hemorrhagic blisters and bullae, marked pain with movement, and overlying erythema of his left lower limb. **(B)** Three days after admission, the area of redness and swelling of the left lower limb gradually spread to the root of the thigh and perineum.

In January, a previously healthy 39-year-old man was brought to our emergency department with a 1-week history of pain and ulceration in his left calf. He developed a sudden pain in the left lower extremity after exercise a week ago, followed by a high fever (40.0 °C) with dizziness, nausea, and shortness of breath. Lower limb pain increased gradually, accompanied by swelling and skin lesions. Twenty-four hours before the visit, the patient showed anuria. Physical examination in the emergency room detected tachycardia (112 bpm) and hypotension (72/39 mmHg). The patient was rapidly admitted to the ICU at the midnight. He was an ex-tobacco smoker for 3 years and drank a little beer sometimes. No other history was recorded, including tumors, organ transplants, immunodeficiency, diabetes, toxic exposure, insect stings, or trauma. Arterial blood gas indicated metabolic acidosis with respiratory alkalosis with a pH of 7.404, and lactic acid of 7.1. His initial blood test results were as follows: white blood cells, 21.33^*^10^9^/L; hemoglobin, 134 g/L; platelets, 44^*^10^9^/L; laboratory studies also showed elevated plasma C-reactive protein levels (541.0 mg/l), while procalcitonin and interleukin-6 (IL-6) levels exceeded the limit of detection (>100 ng/mL and >5000 pg/mL, respectively). Other laboratory tests showed low albumin (20.2 g/L), high serum creatinine (219 μmol/L), elevated serum creatine kinase (1813 U/L), dysfunction of coagulation (INR1.33, PT16.6 s, APTT 66.8 s), and elevated D-dimer (3.7 mg/L). On physical examination, moderate swelling, hemorrhagic blisters and bullae, marked pain with movement, and overlying erythema of his left lower limb were found (Figure 1). Computed tomography (CT) showed diffuse subcutaneous soft tissue lesions in the lower leg and pneumonia. Transthoracic echocardiography demonstrated normal left ventricular function. No abscess was found.

**Figure 2:**
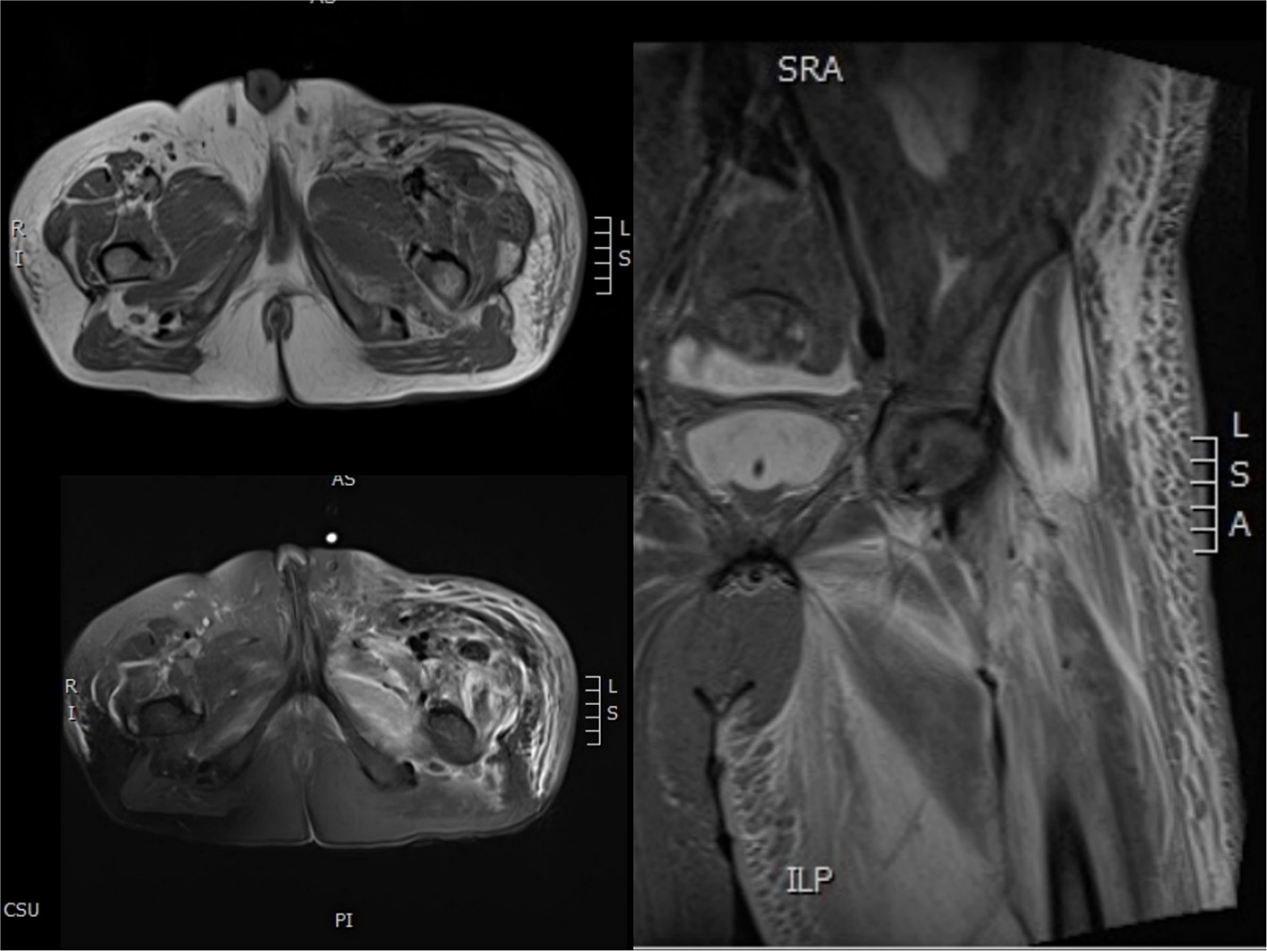
MRI plain scans of the left lower limb.

**Figure 3:**
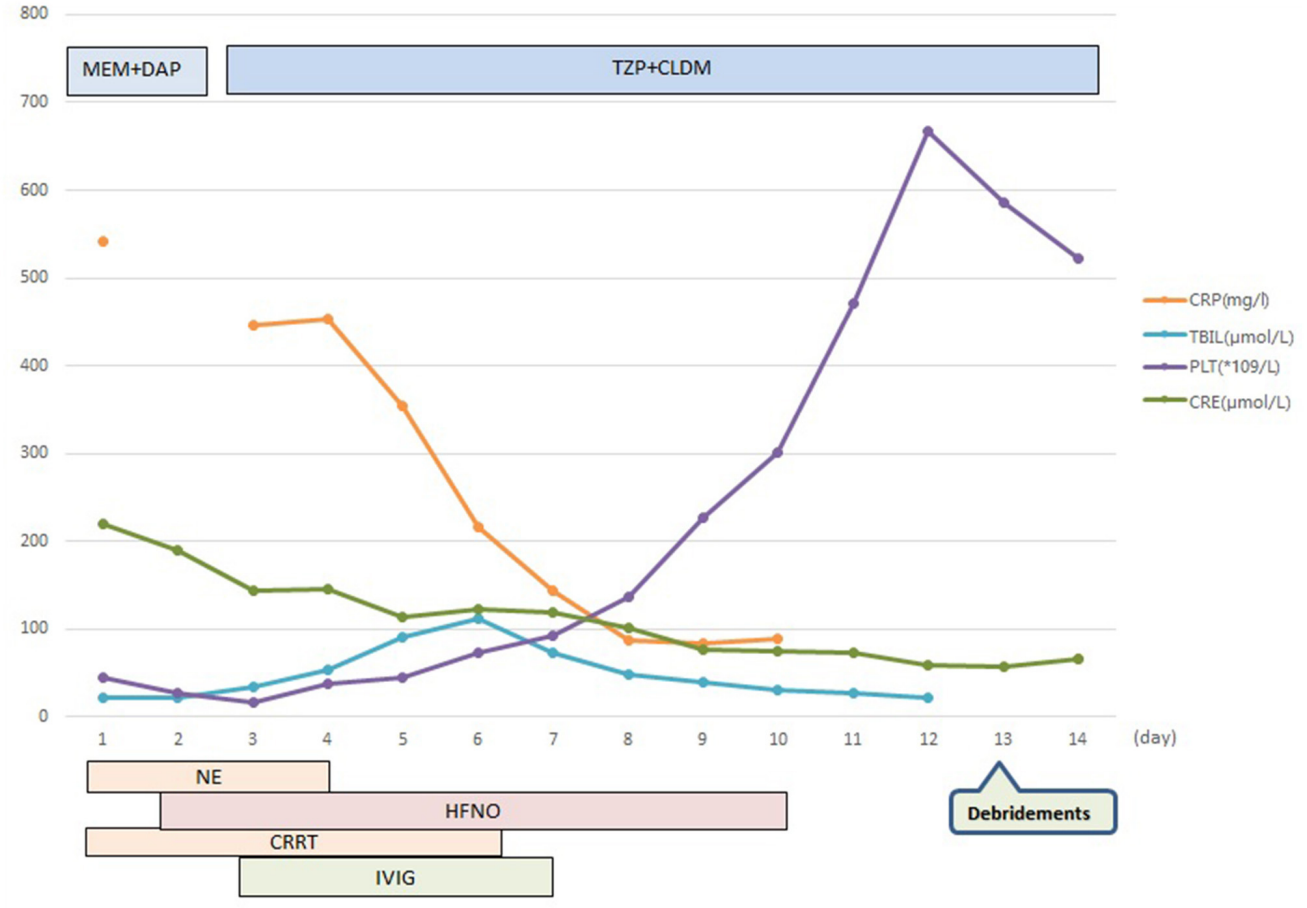
Clinical course with laboratory results and treatment. MEM, meropenem; DAP, daptomycin; TZP, piperacillin/tazobactam; CLDM, clindamycin; CRP, C-reactive protein; TBIL, total bilirubin levels; PLT, platelets; CRE, creatinine; NE, noradrenaline; HFNO, high-flow nasal oxygen; CRRT, continuous renal replacement therapy; IVIG, intravenous immunoglobulin.

The patient was diagnosed with septic shock due to necrosis of the skin and deep soft tissue of the left extremity (2). He immediately received meropenem and daptomycin treatment empirically, fluid resuscitation, and noradrenaline via an internal jugular central venous catheter. Continuous hemodiafiltration was also implied. Although sepsis was diagnosed, the pathogen of the initial infection was unclear. Before the use of antibiotics, cultures, as well as the rapid pathogen detection by metagenomic next-generation sequencing (mNGS), were drawn from the pustule, deep soft tissue (lower limb), and two-site blood samples (both aerobic and anaerobic bottles) at least twice. Cultures were performed by experienced laboratory operators in a standard manner and the mNGS tests were performed following the manufacturer's instructions. Generally, standard samples were handled with quality-filtered, elimination of duplicate readings, removal of low-quality sequences that match human genome sequences. Libraries for mNGS were prepared using the Illumina^®^ DNA Prep, (M) Tagmentation (20018705, Illumina) according to the manufacturer's recommendation. Pooled libraries were sequenced on NextSeq™ 550Dx system using a 75 bp, single-end sequencing kit (Illumina), and at least 20 milslion sequencing reads were acquired for each sample. A negative control sample was processed and sequenced in parallel in each sequencing run for quality control. The data was processed using PIseqTM (Pathogen Identification Sequencing) Metagenomic Sequencing Data Management System V2.0 (WillingMed) simultaneously to draw the conclusion. However, although the patient's hemodynamics was gradually improving after the antibiotic treatment, the area of redness and swelling of the left lower limb gradually spread to the root of the thigh and perineum (Figure 1). Moreover, the patient developed moderate acute respiratory distress syndrome (ARDS) with a PaO_2_/FiO_2_ ratio of 152.5 mmHg and high-flow nasal oxygen (HFNO) was used to improve hypoxemia. There's also liver function damage with total bilirubin levels (TBIL) significantly increased. On the third day after admission, the mNGS results showed GAS both in pustule and deep soft tissue (Table 1). The pustule mNGS report detected a total of 132 unique GAS sequence reads, representing 96.3% of microbial reads and 0.49% of the nucleotide sequence coverage. The soft tissue mNGS report identified a total of 142474 unique GAS sequence reads, representing 100% of microbial reads. The virulence profiling and antimicrobial genes report were also presented (Supplementary Table 1). Thus, we diagnosed this patient as having streptococcal toxic shock syndrome based on the criteria (shown in Supplementary Table 2) (3) and necrotizing fasciitis. Other tests for pathogens such as parasites, epidemic hemorrhagic fever, leptospirosis, tuberculosis were all negative. Therefore, antibiotic therapy comprising piperacillin/tazobactam (4.5g Q6H/day) and clindamycin (900mg Q8H/day) instead of meropenem was given immediately. Because of the low platelet count and disorder of coagulation, the surgeon had to postpone the surgical debridement while observing the tension of the wound closely. Meanwhile, he received intravenous immunoglobulin (IVIG) 0.5g/kg/day for 5 days. Over the next 5 days, his kidney function and coagulation were recovered and the general condition was improved subsequently. The temperature dropped to the normal range, too. Ten days after admission, as the PaO_2_/FiO_2_ ratio increased to 311 mmHg, HFNO could be stopped. At this time, magnetic resonance imaging (MRI) was taken and showed diffuse swelling in bilateral buttock, erector spinal muscle, left thigh root, left lower limb muscle, and muscle space (Figure 2). Three days later, he received the first surgical debridement and then was transferred to the general ward. The patient underwent another debridement surgery and the following skin grafting during the hospitalization. After the operations and rehabilitation exercises, he is now recovering well and ready to be discharged from the hospital. During the patient's stay in the ICU, the results of the cultures were all negative in the blood, pustule, and deep soft tissue. The clinical course with laboratory results and treatments was concluded in Figure 3.

**Table 1:** The results of next-generation sequencing (NGS) of the patient.

**Species name**	**Blood**		**Pustule**		**Deep soft tissue**
	Sequence number (RPTM)^*^	**Relative abundance**	Sequence number (RPTM)^*^	**Relative abundance**	Sequence number (RPTM)^*^
Group A Streptococcus			132	96.3%	142474
Pseudomonas aeruginos	3	100%	6	3.7%	
Mycobacterium tuberculosis complex			1	0.0%	

* *RPTM, Reads per ten million*.

## Discussion and Conclusions

iGAS are defined by the entry of GAS into normally sterile sites with or without clinical evidence of invasive diseases or a deep-seated infection (4). It ranges from mild human infections such as cellulitis to serious infections such as STSS with a high case fatality rate which can reach up to 38% (1). Compared to industrial countries, the fatality rate is even higher in lower- and middle-income regions (5, 6). iGAS affects 663000 people worldwide every year, causing 163000 deaths (7). The incidence of iGAS disease in developed countries varies at 3.8–4.24 cases per 100,000 persons per year (1, 8, 9). Interestingly, data shows that the peak season of iGAS cases usually occur in January and December during the winter months rather than in autumn (8) which is consistent with our case. iGAS can occur in any age and ethnic group, although the incidence is highest in the elderly and young children, and non-white people (1).

Even though iGAS may present in healthy individuals like our patient, specific factors predisposing to the infection have been identified. Skin breakdowns, whether acute or chronic, traumatic or surgical, are the most common risk factors. A lot of other underlying medical conditions are suspected to increase the risk of iGAS (1), including age (in adults 65 years old or older), malignancy, diabetes, injection drug use, viral infections [influenza, varicella, and H1N1 virus pandemic (10)], smoking, immunosuppression, alcohol abuse and other chronic diseases. Although we repeatedly took a detailed history of the patient at the time of admission and after the diagnosis was confirmed, we still did not find any relevant risk factors for our patient. We therefore failed to draw a conclusion about the causative event of his infection. It is worth noting that most patients do not display an identifiable route of infection (11).

The exact mechanism of STSS is not fully clarified. At present, the main pathophysiology of STSS is thought to be associated with the streptococcal toxins expressed by the GAS strain, which function as superantigens and provokes the non-specific proliferation of T-cells (12). This activation then leads to severe cell damage and hyperinflammatory response, resulting in shock and organ failure (13). Despite superantigens including speA, speG, and speJ (14), M-protein is also considered as the major virulence factor of GAS (15). The mNGS virulence profiling confirmed the existence of these related coding genes. The virulence profiling also revealed the genes encoding hyaluronidase, streptokinase, SLO, and DNase, which were thought to be engaged with the invasive ability of GAS as exotoxins and enzymes (16, 17).

The most common clinical syndromes of iGAS are SSTS, isolated bacteremia, and pneumonia. Abrupt and severe pain, usually involving an upper extremity, is the most common initial symptom and the main reason for STSS patients to seek medical help. Up to 20% STSS patients present an influenza-like syndrome characterized by fever, chills, myalgia, and diarrhea (18). Nausea and vomiting accompanied with fever could be seen occasionally. Local swelling and erythema are the most common physical signs at the time of visit. The formation of blisters and bullae, some of which become hemorrhagic, indicates a serious condition, with 70% developing necrotizing fasciitis or myositis requiring surgical debridement or even amputation. It should be noticed that the initial cutaneous appearance may hinder the diagnosis of necrotizing fasciitis. Necrotizing fasciitis should always be considered when the patient presents persistent fever with erythema, heat and induration of the skin and soft tissues. Severe pain and tenderness inconsistent with physical examination is the clinical feature that distinguishes necrotizing fasciitis from superficial infection (19). Rapid progression and expansion of the lesion, paresthesia, bruising, crackles, blistering, or necrosis within a short period will contribute to the diagnosis. Of note, the clinical manifestations of Staphylococcus toxic shock syndrome is similar to STSS, especially for women during menstruation or of any gender who have recently undergone surgery. We have summarized some distinguishing features in Supplementary Table 3.

Laboratory investigation of STSS includes neutropenia, thrombocytopenia, and dysfunction of coagulation or even disseminated intravascular coagulation. Urea nitrogen and creatinine will elevate in patients with renal injury. Rhabdomyolysis or necrotizing fasciitis can lead to markedly elevated creatine kinase levels. The serum levels of inflammatory cytokines also rise. However, none of these tests are specific. Therefore, it is difficult for the emergency staff or inexperienced physicians to diagnose by clinical criteria at the first visit. A high index of suspicion is then required. The possibility of STSS should be in mind for the diagnosis of any patient with severe systemic inflammatory response syndrome of unknown etiology, especially in the case of Gram-positive infections with a relatively minor sauce and a vigorous systemic response. This is particularly important for physicians when diagnostic tools are lacking. Meanwhile, empiric treatment can be initiated whether doubt or not. This patient was considered sepsis at the beginning and active fluid resuscitation was administered following sepsis guidelines without hesitation (20). As for the treatment of STSS, fluid resuscitation is also critical due to severe volume loss and persistent capillary leakage (21). Aggressive therapies to reverse septic shock and maintain organ function bought us time for the follow-up correcting antibiotic strategies.

On the other hand, since STSS often presents severe infection and a high mortality rate, it does require a definite diagnosis as early as possible. Early identification of pathogens may lead to early critical treatment, especially the rational use of antimicrobial agents and prompt debridement. Previous literature demonstrated that blood cultures were positive in majority of patients (about 60%) with STSS (22–24), while cultures from the site of infection had a higher positive rate up to 95%, including tissues taken at surgery, cerebrospinal fluid, synovial fluid, or pleural fluid (18). In our case, the cultures of blood, pustule, and soft tissues were all negative while the mNGS detected the specific pathogen. The mNGS-positive along with culture-negative result may be due to the presence of relic DNA from non-viable GAS or low bacterial load (25). In the biopsies of patients with necrotizing fasciitis caused by iGAS, the mixture of viable and dead bacteria has indeed been confirmed by the use of bacterial viability stain (26, 27).

Confusingly, severe culture-negative cases are rarely reported. Perhaps clinicians don't even realize that these patients should be diagnosed with STSS due to the negative results. There is no doubt that the lack of proper diagnosis and treatment in time will even lead to death. mNGS can serve as a contentment quick tool for identifying a variety of known pathogens or new ones. Compared to traditional culture-based techniques, mNGS only requires a few fragments of the pathogen's DNA obtained directly from patient samples, which makes it more sensitive and precise. In addition, mNGS is also widely used to identify or predict the new antimicrobial resistance gene variants, disease transmission and virulence (28). Interestingly, mNGS is not the only tool for rapid detection. It was reported that an optical immunoassay technology that could detect the GAS antigen within 5–10 min was available (29). Another two rapid molecular tests targeting the *speB* gene of GAS are used experimentally, one of which could get the result in approximately 25 min (30). However, although the *speB* gene is theoretically specific to GAS, the expression could be detected in group C or G Streptococcus (31, 32). This could result in false positive. Herein, mNGS is a more convincible tool for rapid and accurate diagnosis based on clinical symptoms.

Penicillin is still the gold standard for the treatment of iGAS. It has been used for more than 80 years with a low failure rate. Nonetheless, clinical failure of penicillin therapy still existed. In general, beta-lactam antibiotics are believed to be most effective against rapidly growing bacteria. The efficacy may be diminished as organism concentrations increase and the rate of bacterial growth slows. Recently, the adjunction of clindamycin, combined with the traditional beta-lactam antibiotics therapy is extensively adopted. Clindamycin performs well at all stages of bacterial growth (33), which is perfectly compatible with β-lactams to reduce the bacterial load in iGAS. Other potential advantages of clindamycin for the treatment of iGAS include: (a) clindamycin is associated with a longer postantibiotic effect than beta-lactam agents, and (b) clindamycin suppresses the synthesis of penicillin binding proteins, which are involved in cell wall synthesis and degradation (34). Clindamycin also has non-antimicrobial antibiotic effects such as the inhibition of M-protein synthesis, superantigens and other toxins (35). Clindamycin, together with β-lactams, is considered to be effective in the treatment of necrotizing fasciitis or STSS (35, 36). An Australian prospective study suggested that adding clindamycin could reduce mortality (37). What's more, the mNGS report did not show the presence of genes resistant to beta-lactam antibiotics and clindamycin. Therefore, we finally chose the combination of piperacillin/tazobactam (which inhibits cell wall synthesis) and clindamycin (which inhibits protein synthesis) for our patient and the antibiotics proved to be effective.

In addition to proper antibiotics, aggressive surgical intervention is also therapeutically critical in patients with STSS once the site of deep soft-tissue infection has been identified by MRI or CT scans (38). However, what shall we do for those unstable patients who could not perform immediate wide debridements or amputations? In our case, the surgeon made the difficult decision to postpone the surgical debridement because of the patient's poor situation. Such a dilemma is quite common in clinical practice. Prior literature had studied the outcome of those necrotizing fasciitis patients who received the administration of IVIG while without surgery (39). Surprisingly, all included patients were cured well. The effect of antibiotics may also be enhanced by the adjunction of intravenous immunoglobulin (IVIG) simultaneously. Attempts had been made to assess the efficacy of IVIG in the treatment of STSS two decades ago (40). Results showed that IVIG combined with appropriate antibiotics and supportive care could significantly reduce mortality (41). Darenberg et al. further demonstrated that IVIG as adjunctive therapy contributed to improving survival in patients with STSS in 2014 (42). IVIG functions by inhibiting T-cell activation, thereby reducing cytokine release, down-regulating the expression of adhesion molecules, chemokine and chemokine-receptor, and neutralizing superantigens (42, 43). However, although IVIG has been suggested as a possible adjunct, patient outcomes remain varied from different studies. A propensity score-matched study containing 4127 necrotizing fasciitis patients with shock in 130 Unite States hospitals failed to show any impact of IVIG neither on mortality nor length of hospital stay (44). Similar results were obtained in another randomized, blinded, placebo-controlled trial (45). Disappointingly, the result of the trial showed no significant effects of adjuvant IVIG on self-reported physical functioning at 6 months, mortality or organ failure. Despite the controversy, regarding the efficacy and the low risk of side effects, IVIG is still recommended for treatment in patients with unstable hemodynamics and/or having STSS or necrotizing fasciitis (36, 46, 47). Additionally, the timing of administration is crucial because this treatment only offers short-term protection and does not induce active immunity. It should be noted that the optimal dosage or schedule of administration has not been defined. A recent study showed that a 25-g IVIG dose was sufficient to neutralize GAS superantigen activity in plasma (48). Further information on the impact of IVIG therapy in STSS is still required.

In summary, STSS caused by invasive GAS is associated with significant mortality. mNGS could serve as a convincible tool for early and accurate diagnosis based on clinical symptoms. Meanwhile, clindamycin combined with beta-lactam therapy may perform better than single antibiotics. In addition, the use of IVIG combined with antibiotics may offer an alternative medical choice for patients with STSS who are not eligible for aggressive surgical intervention.

## Data Availability Statement

The raw data supporting the conclusions of this article will be made available by the authors, without undue reservation.

## Ethics Statement

Written informed consent was obtained from the individual(s) for the publication of any potentially identifiable images or data included in this article.

## Author Contributions

WH and TT prepared the report manuscript. YZ, BY, and GW contributed to the clinical observations, material preparation, and image collection. TT, CW, and JL interpreted the laboratory results and the MRI and CT images. CW and TT reviewed the manuscript. All authors contributed to the study conception and design and read and approved the final manuscript.

## Funding

The study was supported by the Natural Science Foundation of Hunan Province, China (Grant No. 2021JJ40832 and No. 2019JJ50882), Scientific Research Project of Hunan Provincial Health Commission (202117010086), and Hunan Research Plan of Chinese Traditional Medicine (No. 2021170).

## Conflict of Interest

The authors declare that the research was conducted in the absence of any commercial or financial relationships that could be construed as a potential conflict of interest.

## Publisher's Note

All claims expressed in this article are solely those of the authors and do not necessarily represent those of their affiliated organizations, or those of the publisher, the editors and the reviewers. Any product that may be evaluated in this article, or claim that may be made by its manufacturer, is not guaranteed or endorsed by the publisher.
