# Supplementary material for: Case Report: Therapeutic Strategy With Delayed Debridement for Culture-Negative Invasive Group A Streptococcal Infections Diagnosed by Metagenomic Next-Generation Sequencing
Source: Front Public Health. 2022 May 11;10:899077. doi: 10.3389/fpubh.2022.899077 (PMC9130855; doi:10.3389/fpubh.2022.899077)
Supplement: Supplementary file 1 [file Table_1.docx]

Supplementary Material

# Supplementary Tables

**Table 1. The virulence profiling and antimicrobial genes report of the deep soft tissue mNGS**

| **No.** | **Virulence Factors** | Coding genes [Sequence number] |
| --- | --- | --- |
| 1 | Hyaluronidase | *hylP*[20] |
| 2 | Streptokinase | *ska*[133] |
| 3 | SIC | *sic*[66] |
| 4 | SpeB | *speB*[114] |
| 5 | SLO | *slo*[79] |
| 6 | GRAB | *grab*[44] |
| 7 | M protein | *emm*[90] |
| 8 | IdeS | *ideS/mac*[89] |
| 9 | FBPs | *fbp54*[135]*，fbaA*[80] |
| 10 | C5a peptidase | *scpB*[155]*，scpA*[178] |
| 11 | DNase | *mf/spd*[75]*，mf3*[62]*，mf2*[71] |
| 12 | Hyaluronic acid capsule | *hasA*[121]*，hasB*[134]*，hasC*[92] |
| 13 | pilus | *fctB*[68]，*cpa*[236]，*lepA*[54]，*fctA*[94], *srtC1*[67] |
| 14 | Spes | *spej*[66]，*smeZ*[73]，*ssa*[66]，*spea*[60]， *speg*[61]，*spec*[64] |
| **NO.** | **Antimicrobial Genes** | **Sequence number** |
| 1 | *lmrP* | 60 |

**Table 2. Streptococcal toxic shock syndrome: case definition (CDC 2010)**

| **Hypotension** | Adults: systolic blood pressure≤ 90mmHg | |
| --- | --- | --- |
|  | Children: ≤the fifth percentile by age | |
| **Multi-organ involvement characterized by two or more of the following** | | |
| **Renal impairment** | Adults: Creatinine≥ 2 mg/dL (≥ 177 µmol/L) | |
|  | Children: ≥ twice the upper limit of normal for age | |
|  | Patients with preexisting renal disease: ≥ twofold elevation over the baseline level. | |
| **Coagulopathy** | Platelets≤100,000/mm^3^(≤100*10^6^/L) or disseminated intravascular coagulation (prolonged clotting times, low fibrinogen level, and the presence of fibrin degradation products) | |
| **Liver involvement** | Alanine aminotransferase, aspartate aminotransferase, or total bilirubin levels ≥ twice the upper limit of normal for the patient's age | |
|  | Patients with preexisting liver disease: ≥ twofold increase over the baseline level | |
| **Acute respiratory distress syndrome** | | |
| **Erythematous macular rash** | | may desquamate |
| **Soft-tissue necrosis** | | necrotizing fasciitis or myositis, or gangrene |
| **Case classification** | | |
| **Probable:** |  | |
| Case that meets the clinical case definition in the absence of another identified etiology for the illness and with isolation of group A Streptococcus from a non-sterile site. | | |
| **Confirmed:** |  | |
| Case that meets the clinical case definition and with isolation of group A Streptococcus from a normally sterile site (e.g., blood or cerebrospinal fluid or, less commonly, joint, pleural, or pericardial fluid). | | |
| Source: adapted from the Centers for Disease Control and Prevention. Streptococcal Toxic Shock Syndrome (STSS) (Streptococcus pyogenes) 2010 Case Definition [internet]. [cited 07 Feb 2022.]. Available at: https://ndc.services.cdc.gov/case-definitions/streptococcal-toxic-shock-syndrome-2010/. | | |

**Table 3. Identification of staphylococcal toxic shock syndrome and streptococcal toxic shock syndrome**

| **Features** | **Staphylococcal TSS** | **Streptococcal TSS** |
| --- | --- | --- |
| Bacterial toxins | SEA, SEB, SEC, TSST-1, SEIs | speA, speG, and speJ, M-protein |
| Age | Often in young people | Common in adults＞ 65 years old |
| Host factors | Menstruation, tampon, postpartum, surgical wounds, intrauterine devices, nasal packs | Skin lesions, varicella, diabetes, alcohol abuse, chronic disease, immunosuppression, intravenous drug use, heart disease, cancer |
| Rash | Very common | Less common |
| Desquamation | Very common, particularly of palms and soles, 1 to 2 weeks after onset of rash | Less common |
| Acute pain | Rare | Common and severe, especially inconsistent with physical examination |
| Multisystem involvement | Gastrointestinal, muscular, mucous membranes, renal, hematologic, hepatic, CNS | Renal, coagulopathy, hepatic, acute respiratory distress syndrome, erythematous macular rash, soft-tissue necrosis |
| Progression to multiple organ failure | Very soon | Very soon |
| Laboratory tests | Negative results in Blood, throat, or CSF culture; Negative serologies for Rocky Mountain spotted fever, leptospirosis, or measles | Isolation of group A Streptococcus from a normally sterile or nonsterile site |
| Positive blood cultures | Less than 5% of cases (for S. aureus) | about 60% |
| CNS central nervous system; CSF cerebrospinal fluid; SEs staphylococcal enterotoxins; SEIs staphylococcal enterotoxin-like toxins; SPE superantigen pyrogenic exotoxins; TSS toxic shock syndrome; TSST-1 toxic shock syndrome toxin 1 | | |
| Source: adapted from the Centers for Disease Control and Prevention. Toxic Shock Syndrome (Other Than Streptococcal) (TSS) 2011 Case Definition [internet]. [cited 09 Apr 2022.]. Available at: https://ndc.services.cdc.gov/case-definitions/toxic-shock-syndrome-2011/. | | |
